# Differentiable Resolution Allocation for Tiny Object Detection in UAV Imagery

**DOI:** 10.3390/s26196157

**Published:** 2026-09-28

**Authors:** Ergashevich Halimjon Khujamatov, Khasanov Doston, Yusupov Sarvarbek Sodikovich, Oybek Usmankulovich Mallaev, Shakhnoza Muksimova, Alisher Mamatov, Jinsoo Cho, Razvan Craciunescu

**Affiliations:** 1 Department of Computer Engineering, Gachon University, Seongnam-si 13120, Gyeonggi-go, Republic of Korea; kh.khujamatov@tuit.uz (E.H.K.); shakhnoza02@gachon.ac.kr (S.M.); 2 Department of Data Communication Networks and Systems, Tashkent University of Information Technologies, Tashkent 100084, Uzbekistan; dhasanov@tuit.uz; 3 Department of Mechanical Engineering Kimyo International University in Tashkent, Tashkent 100121, Uzbekistan; s.yusupov@kiut.uz; 4 Department of Digital Technologies, Alfraganus University, Yukori Karakamish Street 2a, Tashkent 100190, Uzbekistan; o.mallayev@afu.uz; 5 Department of Digital Pedagogy and Media Education Development, Republican Scientific and Methodological Center for the Development of Education of the Republic of Uzbekistan, Tashkent 100011, Uzbekistan; a.mamatov1991@gmail.com; 6 Department of Telecommunications, Faculty of Electronics, Telecommunications and Information Technology, National University of Science and Technology POLITEHNICA Bucharest, 060042 Bucharest, Romania

**Keywords:** differentiable crop selection, learned resolution allocation, foveated object detection, discrete wavelet transform, Gumbel top-K sampling, density-conditioned queries, aerial imagery, edge deployment

## Abstract

**Highlights:**

**What are the main findings?**

- On the VisDrone2019 validation set, AFWD-Net reaches 49.3% mAP@0.5, 30.4% mAP, and 22.8% *AP_s_*, against 35.2%, 19.6%, and 11.8% for the YOLOv8-s baseline (11.2 M parameters) under an identical evaluation protocol. The 11.0-point improvement in *AP_s_* nearly doubles the baseline’s small-object accuracy and stems chiefly from the proposed adaptive foveation, with further contributions from wavelet detail recovery and density-conditioned queries.
- Learning where to allocate high-resolution computation jointly with the detection objective outperforms both uniform-resolution detectors and fixed, non-differentiable tiling, at a moderate 15.7 M parameters, and a distilled 7.2 M variant still surpasses all prior methods.

**What are the implications of the main findings?**

- Accurate detection of targets under 32 × 32 pixels is achievable within the latency and memory budget of embedded UAV accelerators, making real-time onboard perception practical for traffic monitoring, precision agriculture, inspection, and search and rescue.
- Restoring high-frequency edge information lost to backbone downsampling, rather than adding model capacity, is the key lever for tiny object recognition, suggesting frequency-domain recovery and learned resolution allocation as general design principles for aerial detectors.

**Abstract:**

Aerial scenes exhibit a mismatch between where computation is spent and where information resides: targets occupy a small, unevenly distributed fraction of the frame, yet detectors process every region at identical resolution. Tiling and clustering pipelines exploit this sparsity, but the rule deciding which regions deserve magnification is hand-designed and never observes the detection loss. AFWD-Net removes that separation. A coarse density estimator, run on a heavily downsampled copy of the frame at negligible cost, produces a spatial prior over likely target locations; Gumbel top-K relaxation and a spatial-transformer sampler convert this prior into a small set of high-resolution crops whose centers and scales are differentiable, so the gradient that measures detection quality also determines where resolution is allocated. Within each crop, a learnable wavelet decomposition separates approximation from directional detail sub-bands and enhances the latter under edge supervision confined to annotated regions, restoring the high-frequency structure that stride downsampling erases, precisely the cue separating a genuine sub-32-pixel target from background clutter. Detection queries are seeded from the same density field rather than a fixed lattice, and crop-level predictions are merged by density-calibrated soft suppression. On VisDrone2019-DET the model attains 49.3% mAP@0.5, 30.4% mAP@[0.5:0.95] and 22.8% APS at 15.7 M parameters; a distilled 7.2 M student remains competitive with all compared methods. Experiments on UAVDT, AI-TOD-v2 and SODA-A assess cross-dataset generalization.

## 1. Introduction

UAVs have become a routine sensing platform for urban traffic monitoring, precision agriculture, infrastructure inspection, environmental surveying, and search and rescue operations [1]. In nearly all of these applications the objects of interest—vehicles, people, livestock, defects, and debris appear small relative to the frame because they are imaged from altitude and at oblique angles. A large fraction of these targets falls within what the literature defines as the small and tiny object regime: objects occupying fewer than 32 × 32 pixels, less than roughly one percent of the total image area. At these scales the accuracy of otherwise strong detectors degrades sharply, and closing this gap is widely regarded as one of the defining challenges of aerial computer vision [2]. Here are three difficulties that are compounded in the UAV setting. First, limited pixel coverage yields impoverished feature representations a target only a handful of pixels across simply does not contain enough signal for a deep network to form a discriminative descriptor [3]. Second, the successive stride convolutions and pooling layers that give modern backbones their large receptive fields also progressively destroy the fine, high-frequency detail on which small-object recognition depends, so that by the deeper stages of the network a tiny object may have no distinct spatial footprint at all [4]. Third, objects in aerial scenes are distributed unevenly; they cluster densely in some regions and are absent from large background areas, and occlusion within these clusters tends to be locally concentrated rather than randomly scattered, producing feature confusion between neighboring instances [5]. The value of a UAV detector is realized only when it runs onboard, in real time, on an embedded accelerator with a fixed power and memory envelope [6]. This rules out the brute force remedy of simply processing every frame at full resolution, and it penalizes architectures whose cost scales with image area rather than with the number of objects actually present. Relatively few published methods jointly address the extreme scale accuracy problem and the embedded-efficiency problem; most improve one at the expense of the other [7].

Existing strategies fall into two broad families. Uniform-resolution detectors, typically YOLO family and transformer-based models, augmented with multi-scale necks, attention modules, and dedicated shallow prediction heads improve feature quality but still expend the same computation on empty background as on target-rich regions [8]. Crop-based tiling detectors instead partition the frame and run inference on sub-images, which recovers effective resolution but introduces a separate, hand designed and non differentiable preprocessing stage the tiling grid is fixed, it dices background as finely as foreground, and its boundaries create duplicate and truncated detections that must be cleaned up heuristically. In both families the decision of where to spend resolution is divorced from the detection loss [9]. Object detection has progressed considerably over the past decade. Early gains came from deep convolutional networks, after which region proposal frameworks such as Faster R-CNN set a strong accuracy benchmark [10]. More recent work has pushed in two complementary directions efficient one-stage detectors like YOLOv4, which prioritize speed, and transformer based formulations such as DETR, which reframe detection as a set-prediction problem. Despite this progress, aerial imagery continues to pose distinct difficulties [11]. Scenes captured by UAVs are typically populated by objects that are small, densely clustered, and subject to wide variations in scale, all of which strain detectors designed for conventional ground-level views [12]. This difficulty is compounded by the increasing sophistication of aerial platforms themselves; recent UAV research highlights the corresponding demand for perception systems that are both robust and efficient enough to run onboard. Taken together, these constraints point toward adaptive detection strategies that concentrate computation on informative regions rather than expending equal effort across the entire image [13].

We argue that this decision should itself be learned. This paper introduces AFWD-Net, an architecture organized around two coupled mechanisms. The first is an adaptive foveation module a very lightweight density estimator predicts a coarse map of likely small object locations, and a differentiable selector uses that map to choose a small set of variable scale, high-resolution crops. Because gradients flow from the detection loss back through the selection, the network learns to place its limited high-resolution budget exactly where it improves accuracy an artificial analog of the way the human eye saccades to salient regions. The second mechanism is a wavelet detail recovery branch that decomposes each crop into low and high frequency sub-bands, enhances the high-frequency components under edge supervision, and reconstructs the crop before detection, directly counteracting the detail loss caused by downsampling. The contributions of this work are as follows:

- We propose a differentiable adaptive-foveation mechanism that couples a cheap density prior to a learned, resolution allocating crop selector, unifying the previously separate stages of region selection and detection under a single objective.
- We introduce a wavelet detail recovery branch that restores high-frequency edge information destroyed by backbone downsampling, targeting the specific mechanism responsible for tiny object feature degradation.
- We design a density conditioned sparse detection head whose query placement adapts to predicted object density, mitigating the missed detections that arise when fixed anchors and grids fall between clustered targets.
- We specify a complete, reproducible evaluation protocol across four standard aerial benchmarks together with an on-device efficiency assessment, and we release configuration and ablation details to support fair comparison.

The remainder of the paper is organized as follows. Section 2 reviews related work on small-object detection, density and crop guided aerial detection, and frequency-domain enhancement. Section 3 details the AFWD-Net architecture and its training objective. Section 4 describes the datasets, metrics, implementation, and experimental protocol and reports results. Section 5 discusses findings, limitations, and threats to validity, and Section 6 concludes.

## 2. Related Works

Research relevant to AFWD-Net spans four largely separate lines of work, each addressing one facet of the small-object detection problem while leaving the others open. We review them in turn. Section 2.1 surveys the general problem of small and tiny object detection, establishing the standard definitions, difficulties, and benchmarks that frame the task. Section 2.2 examines detectors specialized for UAV and aerial imagery, which improve feature representation and efficiency but operate at a single, uniform resolution. Section 2.3 covers density and crop guided methods, which spend computation non-uniformly by detecting within object-dense regions, yet decouple that region selection from the detection objective. Section 2.4 reviews frequency domain and wavelet approaches, which recover the fine detail lost to downsampling but apply this recovery globally rather than where resolution has been concentrated and positions the present work with respect to all three, motivating the unified design that couples differentiable, detection-driven foveation with region-localized wavelet detail recovery. Throughout, we emphasize that these lines are complementary rather than competing AFWD-Net is designed to combine their respective strengths within a single objective.

### 2.1. Small and Tiny Object Detection

The detection of small and tiny objects has matured into a distinct sub-field with its own surveys, taxonomies, and benchmarks [14]. Recent comprehensive reviews converge on a consistent diagnosis of why the problem is hard objects that span only a handful of pixels carry too little appearance information to form discriminative features, the repeated downsampling of deep backbones progressively erases what detail does exist, scale imbalance between small and large instances skews training, and small targets frequently fall outside the positions covered by predefined anchors and grid cells [15]. Surveys focused specifically on aerial imagery add that the airborne viewpoint compounds these effects through extreme object density, wide scale variation within a single frame, and cluttered backgrounds [16]. A parallel line of reviews has begun to chart newer directions such as open vocabulary detection from UAV platforms, signaling that the community is expanding beyond closed-set accuracy toward generalization and semantic flexibility [17]. Across this literature the remedies proposed for the core accuracy problem fall into recognizable groups, higher-resolution feature pyramids, super-resolution, feature reconstruction branches, refined label assignment and sampling schemes, and loss functions less sensitive to small localization errors, but these operate within a uniform-resolution pipeline and do not change how computation is distributed across the image [18]. The definitions themselves are now standardized the small regime is commonly delimited at an area below 32 × 32 pixels, while dedicated tiny object benchmarks push the mean object size down to roughly a dozen pixels, and aerial small-object benchmarks further stratify the sub-1024-pixel range into finer bands [19]. These definitions and benchmarks frame the regime in which AFWD-Net operates and against which it is evaluated [20].

### 2.2. Detectors for UAV and Aerial Imagery

A large and fast-growing body of work adapts general-purpose detectors to the aerial domain, most visibly through modifications of the YOLO family. The dominant strategy is to strengthen multi-scale representation and add shallow, high-resolution prediction heads so that small targets survive to the output stage. Representative recent detectors introduce multi-scale feature fusion schemes and dedicated tiny object heads built on modern YOLO backbones [12], integrate attention mechanisms across channel and spatial dimensions to sharpen feature extraction in cluttered scenes [14], and redesign backbone and neck modules to preserve fine-grained information through the network [15]. A second and equally active thread prioritizes efficiency for onboard deployment, proposing lightweight networks that trim parameters and computation while retaining small-object recall, often through enhanced but economical feature fusion [21]. This efficiency focus extends to specialized sensing conditions, including infrared UAV imagery [10] and time-critical search and rescue, where detectors must run in real time on civil agency hardware [9]. Application-driven studies of this kind repeatedly confirm the same three obstacles small target size, dense and occluded arrangements, and limited onboard computer and treat them as the defining constraints of the task [22]. Beyond the convolutional YOLO lineage, transformer and query-based detectors have been adapted to aerial scenes, with recent designs manipulating object queries and decoder features according to predicted object density to improve efficiency and small-object recall [18,19]. These methods collectively advance the accuracy efficiency frontier of single-resolution inference, and AFWD-Net is complementary to them rather than competing any such detector can serve as the per-crop backbone within our foveated pipeline, inheriting its improvements while gaining the resolution-allocation mechanism these architectures lack.

### 2.3. Density and Crop-Guided Aerial Detection

To recover effective resolution without paying the cost of processing an entire high-resolution frame, a distinct family of methods spends computation non-uniformly by first locating object-dense regions and then detecting within them. Cluster and density-guided pipelines estimate where objects concentrate and crop those regions for a second, higher-resolution detection pass, while slicing-based inference schemes tile the frame with overlap and fuse the per-tile results [22]. Efficient high-resolution detectors in this vein explicitly route computation toward promising areas and report substantial FLOP savings relative to dense inference [22]. Related work improves the sample selection and feature learning side of the same problem, for instance by selecting more informative training samples for small objects by purifying and relabeling noisy small-object annotations to strengthen supervision [23,24]. This body of work establishes the central premise on which AFWD-Net builds, that resolution should be allocated where objects are, not uniformly, but it also exposes the limitation we target. In these approaches the region selection stage is typically a separate component the tiling grid is fixed and dices background as finely as foreground, the clustering rule is hand-tuned, and the selector, when learned at all, is trained against a proxy objective such as annotation density rather than against detection accuracy itself [25]. Crop boundaries additionally introduce duplicate and truncated detections that must be cleaned up heuristically [26]. AFWD-Net departs from this family precisely at the selection stage by making crop centers and scales the output of a differentiable operation trained end-to-end with the detector, the allocation of resolution is optimized directly for detection quality, and boundary effects are resolved by a density-aware re-assembly step rather than by a fixed post hoc rule [27]. Methods of this kind primarily target semantic confusion among closely related classes AFWD-Net, by contrast, addresses information loss arising from the extremely small spatial footprint of aerial targets by using adaptive resolution allocation and localized high-frequency recovery. The two directions are thus complementary, one resolving what an object is when classes look alike, and the other recovering whether a target is even discernible at very low resolution.

### 2.4. Frequency-Domain and Wavelet Methods in Vision

A growing literature exploits frequency-domain analysis to counteract the detail loss that afflicts small-object detection. The wavelet transform is central to this trend because it decomposes an image and feature map into a low-frequency approximation and directional high-frequency sub-bands, separating global structure from the fine edges and textures that distinguish tiny objects from background. Recent detectors and downsampling modules use learnable wavelet transforms as information-preserving replacements for strided convolution and pooling, retaining detail that conventional downsampling discards [28]. Haar wavelet attention and enhancement modules have been shown to improve remote sensing detection while keeping parameter counts low [27], and Texture-Reconstructive downsampling has been proposed specifically for multi-scale object detection in UAV imagery [26]. Frequency-aware frameworks have also been designed to preserve high-frequency edge and texture cues under degraded conditions such as fog and low light, where those cues are most vulnerable [29], and frequency decoupling has been integrated into query-based aerial detectors [20]. Complementary approaches attack the same detail loss problem from the spatial side, for example by reconstructing a difference map that highlights weak tiny object signals by designing feature pyramids that jointly perceive high-frequency and spatial context [30]. These methods demonstrate the value of explicitly modeling high-frequency information, but they generally apply frequency processing uniformly across the whole feature map and backbone [31]. AFWD-Net instead concentrates a wavelet detail-recovery branch inside each adaptively selected crop and supervises it with an edge consistency term localized to object regions, so that the enhancement is directed at the boundaries that matter for tiny object discrimination rather than applied indiscriminately across the frame [32].

Uniform-resolution UAV detectors improve feature quality but spend computation evenly regardless of where objects lie [33] density and crop-guided methods spend computation non-uniformly but decouple region selection from the detection objective [21,22,23,24] and frequency-domain methods recover lost detail but do so globally rather than where resolution has actually been concentrated [34]. AFWD-Net unifies the strengths of these three lines while addressing their respective gaps it makes resolution allocation differentiable and trains it jointly with detection, it restores high-frequency detail selectively within the regions that allocation identifies, and it conditions detection query placement on predicted object density [35,36]. To our knowledge, the combination of a differentiable, detection-driven foveation mechanism with region-localized wavelet detail recovery in a single objective has not previously been reported for small-object detection in aerial imagery.

## 3. Proposed Method

**Figure 1:**
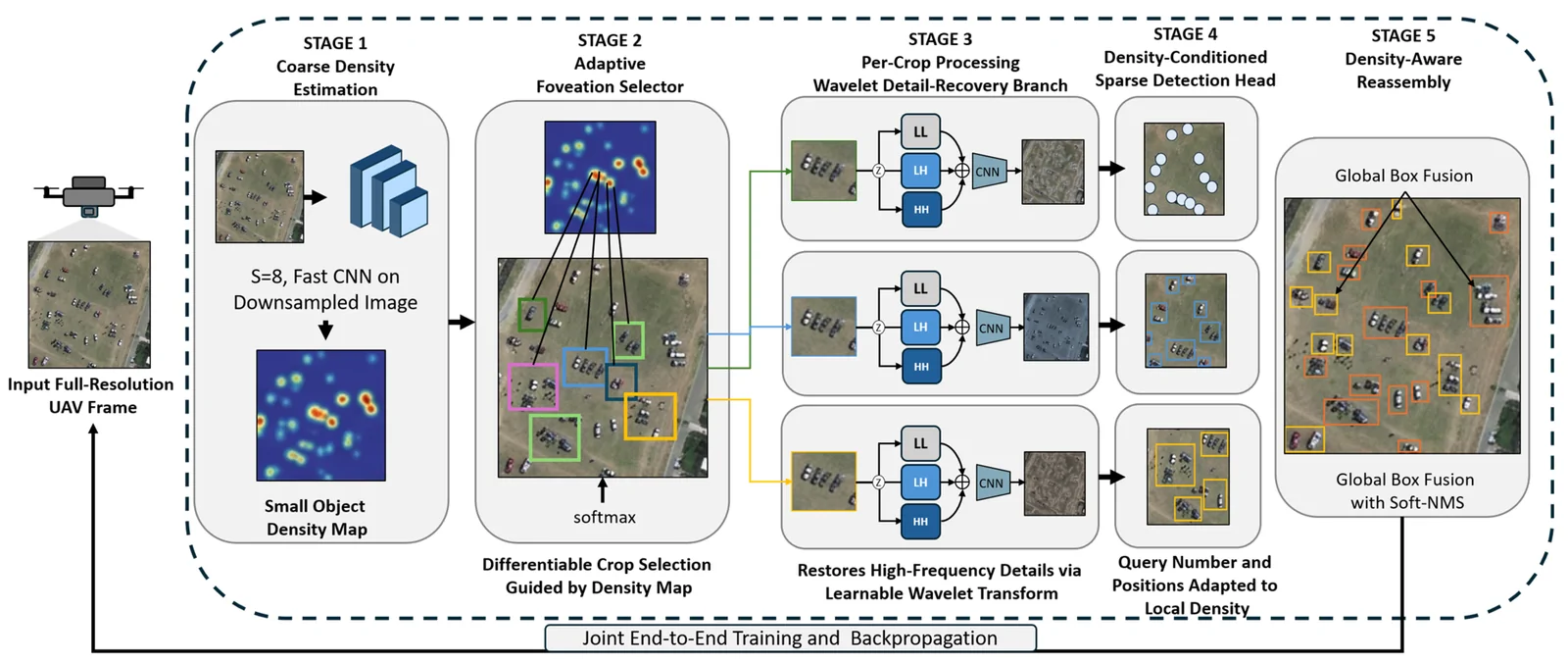
Overall architecture of the proposed AFWD-Net for tiny object detection in UAV imagery.

AFWD-Net processes a full-resolution UAV frame through five stages. (i) A coarse density estimator operating on a heavily downsampled copy of the frame predicts a small-object density map at negligible cost. (ii) An adaptive foveation selector converts this map into a small set of high-resolution crops through a differentiable sampling operation. (iii) Within each crop, a wavelet detail-recovery branch restores high-frequency detail before feature extraction. (iv) A density-conditioned sparse detection head, whose query budget is initialized from the density map, produces per-crop detections. (v) A density-aware re-assembly step maps crop-level prediction back to global image coordinates and resolves overlaps. The entire pipeline is trained with a single composite objective, so the density estimator, the selector, the recovery branch, and the detector are optimized together. Figure 1 summarizes the architecture.

### 3.1. Coarse Density Estimation

Detection requires precise localization and classification and is therefore expensive; a spatial prior over object occurrence requires neither and can be computed on a heavily downsampled image at a small fraction of the detector’s cost. By decoupling the where-to-look question from the what-is-there question, AFWD-Net obtains an image-specific attention prior that drives the foveation selector while adding only marginal overhead. The estimator is deliberately kept coarse; over-investing here would defeat its purpose, and any localization error it introduces is corrected downstream by the high-resolution detection head operating on the selected crops. Let $I\in R^{\left(H\times W\times 3\right)}$ denote the input frame. We form a downsampled copy $I↓\in R^{\left(\frac{H\times W}{s}\times 3\right)}$ at stride s (default s = 8) using area-based resampling, which preserves aggregate intensity better than nearest-neighbor subsampling and suppresses aliasing of the very small structures we care about. A compact fully convolutional network $f_{den}$ with parameters $\theta_{den}\;$ maps I↓ to a single channel density response:

(1) $$D=\;\sigma \left(f_{den}\left(I↓;\;\theta_{den}\right)\right)\;\in {\left[0,1\right]}^{\left(\frac{H\times W}{s}\right)}$$

where *σ* is the logistic function applied per pixel, so each location holds a probability-like score that a small-object center projects onto that cell of the coarse grid. We write $D_{xy}\;$ for the score at coarse grid location. $f_{den}$ is intentionally lightweight. In the default configuration it is a five-stage encoder light decoder: three depth-wise separable convolution blocks with channel widths 16, 32 and 64 extract features at strides ×2, ×4, and ×8 relative to I↓, a single 1×1 bottleneck fuses them, and a two-layer head with one bilinear upsampling step returns the response to the H/s × W/s grid before the sigmoid. There are no residual bottlenecks, attention layers, or feature pyramid necks; the map is coarse by design, and its supervision (below) does not reward fine spatial precision. Because the input is already at 1/s scale and the widths are small, the module contributes on the order of a few hundred thousand parameters and a negligible share of total FLOPs relative to the per-crop detector, which is what makes the estimate-then-foveate strategy net-positive rather than an added burden.

The target map D* is built from the box annotations by rendering a 2D Gaussian at each object center and compositing overlapping Gaussians by element-wise maximum, following the key point heatmap paradigm. For an object i with center in coarse grid coordinates and a bandwidth $p_{i}$,

(2) $$D_{xy}={max}_{i}exp\left(\frac{-\left[{\left(x-u_{i}\right)}^{2}+{\left(v_{i}-y\right)}^{2}\right]}{{2p}_{i^{2}}}\right)$$

The choice of bandwidth $p_{i}$ is the one design decision that materially affects behavior, and it must be adaptive to object size rather than fixed. A single global ρ would either smear tiny objects into the background or leave larger objects with a near-degenerate one-pixel target, and in dense clusters a too-wide kernel merges neighboring instances into an indistinct blob, precisely the confusion we are trying to avoid. We therefore set the kernel width to scale with the square root of the object area in the original image, divided by the stride to convert it into coarse-grid units:

(3) $$p_{i}=clip\left(\frac{\gamma \times \sqrt{\left(w_{i}h_{i}\right)}}{s,p_{min},p_{max}}\right)$$

The scale factor γ and the clip bounds $p_{min},p_{max}\;$ are hyperparameters, small objects, and thus receive tight, well-separated peaks, while larger objects receive proportionally broader support. Using area rather than a per-axis width also keeps highly elongated boxes from producing a streaked target that biases the peak location.

The estimator is trained with a penalty reduced focal loss, which addresses the extreme foreground background imbalance of the density maps; the overwhelming majority of coarse grid cells are background, and a plain binary cross-entropy would be dominated by them. Writing α and β for the focusing exponents and N for the number of object centers in the frame,

(4) $$L_{den}=-\frac{1}{N}\sum_{x,y}\{\begin{array}{cc} {\left(1-D_{xy}\right)}^{\alpha }log\left(D_{xy}\right), & if\;D_{xy}^{*}=1\\ {\left(1-D_{xy}^{*}\right)}^{\beta }{\left(D_{xy}\right)}^{\alpha }log\left(1-D_{xy}\right), & otherwise \end{array}$$

Two mechanisms operate here. The ${\left(1-D_{xy}\right)}^{\alpha }$ term downweights already-confident positive predictions, so training focuses on hard, still uncertain peaks. The ${\left(1-D_{xy}^{*}\right)}^{\beta }$ term applies a soft penalty reduction to negatives cells lying in the Gaussian skirt around a true center are penalized less than cells far from any object, because a near miss on the coarse grid is not a true error the exact center is recovered later at high resolution. Normalizing by N keeps the loss scale stable across frames with wildly different object counts, which is common in aerial scenes. Typical values are α = 2 and β = 4, though these should be confirmed empirically for your data.

### 3.2. Adaptive Foveation Selector

**Figure 2:**
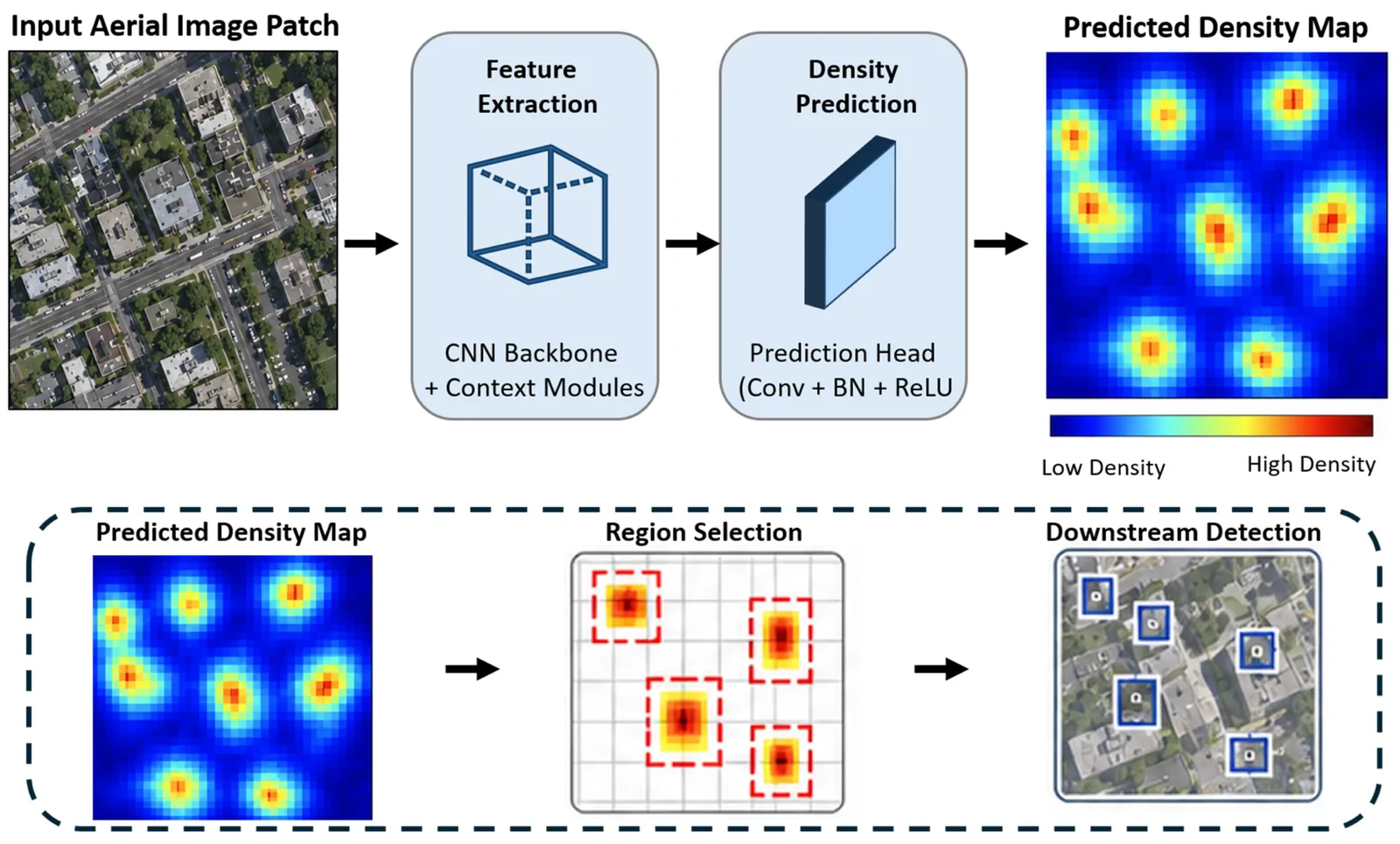
Density-guided candidate generation and crop selection.

The selector is where AFWD-Net departs most sharply from prior crop-based aerial detectors. In slicing, cluster-based pipelines the region selection stage is a fixed heuristic—a regular grid, a clustering rule tuned offline and it is trained, if at all, against a proxy target rather than against detection quality itself. This creates two problems: the selector cannot learn that some high-density regions are already easy and need no extra resolution while some sparse regions contain the hardest targets, and its errors are invisible to the detector’s gradient. Our design removes the barrier the crop centers and scales are produced by a differentiable operation, the crops are extracted by a differentiable sampler, and the detection loss therefore propagates all the way back to the selection. The network learns where to spend resolution because misplaced crops are penalized by the very loss they degrade. The biological analogy is deliberate the mechanism is an artificial foveation, allocating a small high-acuity budget to salient regions, much as the eye saccades to points of interest (Figure 2).

The selector does not search the whole image continuously; it refines a discrete set of candidates seeded from the density prior. From the density map D of §3.2 we extract the M strongest local maxima by non-maximum suppression on the coarse grid, with M > K so the selector has more candidates than it will ultimately keep (default *M* = 4*K*). Each candidate *m* is described by a coarse grid center, a score the density mass integrated over a fixed window $W_{m}$ around the peak, and an initial scale estimate derived from the spatial spread of *D* within that window:

(5) $$s_{m}=\sum_{\left\{\left(x,y\right)\in W_{m}\right\}}D_{xy}$$

Seeding from *D* rather than from a dense anchor lattice is what keeps the candidate set small and the operation cheap; the peaks already concentrate on plausible object regions. Because the density map is coarse, the seeded center and scale are only approximate. A tiny prediction head $f_{ref}$ outputs a sub-grid center offset and a log-scale correction Δlog $\sigma_{m}$, giving the refined parameters with the multiplication by stride s returning the center to full-resolution coordinates:

(6) $$c_{m}=\left(u_{m}+∆u_{m}\right)\times s,\;\sigma_{m}={\hat{\sigma }}_{m}\times exp\left(∆log\sigma_{m}\right)$$

Selecting K candidates from the M proposals is a discrete operation and therefore blocks direct gradient propagation. We implement it with a straight-through Gumbel top-K estimator using sampling without replacement. Let $a_{m}$ denote the score of candidate m. At selection step k, Gumbel noise $g_{m}^{\left(k\right)}$ is added only to candidates not chosen in earlier steps, and the corresponding relaxed probability is

(7) $$p_{m}^{\left(k\right)}=\frac{exp\left(\frac{a_{m}+g_{m}^{\left(k\right)}}{\tau }\right)}{\sum_{j\in R_{k}exp\left(\frac{a_{j}+g_{j}^{\left(k\right)}}{\tau }\right)}},\;m\in R_{k}$$

where τ is the Gumbel softmax temperature, and $R_{k}$ denotes the set of candidates remaining after the first k − 1 selections. The hard forward selection is $h^{\left(k\right)}=onehot\left(arg{max}_{m}p_{m}^{\left(k\right)}\right).\;$During backpropagation, we use the straight-through estimator $z^{\left(k\right)}=h^{\left(k\right)}+p^{\left(k\right)}-stopgrad\left(p^{\left(k\right)}\right).$ Each selected candidate is turned into a high-resolution crop by a spatial-transformer sampling operation. From the center $c_{k}$ and scale $\sigma_{k}$ we construct an affine sampling grid mapping normalized output coordinates to input coordinates, and bilinearly sample the full-resolution frame on that grid to obtain a fixed-size crop:

(8) $$C_{k}=sample\left(I,\tau \left(c_{k},\sigma_{k}\right)\right)\;\in R^{\left(h_{c}\times w_{c}\times 3\right)}$$

Bilinear sampling is differentiable with respect to the grid, and hence with respect to $c_{k},\sigma_{k}$, so the chain of gradients from the detection loss reaches the refinement head and the density estimator. Resampling all crops to a common $h_{c}\times w_{c}\;$ means each is presented to the per-crop backbone at an effectively magnified, normalized scale; a small distant object and a larger nearby one are both delivered at a workable resolution which is the concrete way the selector recovers effective resolution without processing the whole frame at full scale.

Left unconstrained, a purely detection driven selector could degenerate: it might pile several crops onto the single easiest cluster it might drift crops to overlap redundantly. A coverage term rewards the selected crops for jointly explaining the density mass, and an overlap term penalizes redundant intersection:

(9) $$L_{sel}=-\sum_{x,y}D_{xy}\times \begin{array}{c} max\\ k \end{array}m_{k}\left(x,y\right)+\lambda_{ov}\sum_{k\neq l}area\left(C_{k}⋂C_{l}\right)$$

where $m_{k}\left(x,y\right)\in \;[0,\;1]$ is the soft footprint of crop k on the image plane and $\lambda_{ov}\;$ controls the overlap penalty. The first term is maximized when every high-density location is covered by at least one crop; the max over k means a location only needs to be claimed once, so the term does not reward piling crops on the same spot. The second term discourages wasteful double coverage while still permitting the modest overlap needed so that objects straddling a crop boundary are seen whole by at least one crop. Together they spread the resolution budget across the target rich regions rather than concentrating it.

Candidate centers near the image border can generate sampling grids that extend outside the frame; we clamp $c_{k}$ so the crop stays within I, adjusting $\sigma_{k}$ rather than letting the sampler read zero padded regions that would inject spurious edges into the wavelet branch. When the density map is nearly empty, a frame with few and no small objects, the top K scores fall below a confidence floor and the selector falls back to a small set of evenly spaced default crops, guaranteeing the detector always receives valid input and preventing silent failure on out of distribution scenes.

### 3.3. Wavelet Detail-Recovery Branch

Within each crop $C_{k}$, an early feature map F is decomposed by a learnable discrete wavelet transform (DWT) into four sub-bands: a low-frequency approximation $F_{LL}$ and three high-frequency components $F_{LH},F_{HL},F_{HH}$ capturing horizontal, vertical, and diagonal detail. A lightweight high-frequency enhancement module $g_{hf}\;$ refines and denoises the high-frequency bands, after which the inverse transform (IDWT) reconstructs an enhanced feature map:

(10) $$\left(F_{LL},F_{LH},F_{HL},F_{HH}\right)=DWT\left(F\right),\;\hat{F}=IDWT\left(F_{LL},g_{hf}\left(F_{LL},F_{LH},F_{HL},F_{HH}\right)\right)$$

To direct the enhancement at object boundaries rather than at arbitrary texture, we add an edge consistency loss computed only inside ground truth boxes, comparing the gradient magnitude of the reconstructed crop with that of a high-resolution reference:

(11) $$L_{wav}=\frac{1}{\left|\Omega \right|}\sum_{\left(x,y\right)\in \Omega }{\left\|\nabla {\hat{C}}_{xy}-\nabla C_{xy}^{*}\right\|}_{1}$$

where Ω is the union of object regions, Ĉ is the reconstructed crop and C* the reference. This recovers the fine edges that strided downsampling would otherwise erase, which are the cues most useful for separating a genuine tiny object from background clutter.

Rather than tiling the crop with a fixed anchor grid, a fixed number of queries, we initialize the number and coarse positions of object queries from the density map restricted to the crop. Query positions are seeded at density peaks and refined by a query-based decoder, so that detection capacity is concentrated where objects are predicted to be and is not wasted on empty regions, directly addressing the failure mode in which clustered small objects fall between fixed anchor centers. The detection loss combines classification, localization, and a repulsion term:

(12) $$L_{det}=L_{cls}\left(focal\right)+\lambda_{box}L_{CIoU}+\lambda_{rep}L_{rep}$$

The repulsion term $L_{rep}$ penalizes predicted boxes for distinct instances that drift toward one another, countering the locally clustered occlusion characteristic of aerial scenes and reducing duplicate suppression errors in dense groups.

Predictions from all crops are mapped back into global frame coordinates using the inverse of each crop’s sampling grid. Because crops may overlap and their boundaries may bisect objects, we replace hard non-maximum suppression with a density-aware soft NMS in which each detection’s confidence is calibrated by the local density score before overlap resolution. This suppresses duplicate detections at crop seams while preserving genuine neighboring instances in dense clusters. All components are trained jointly by minimizing the weighted sum of the stage losses:

(13) $$L=\lambda_{1}L_{den}+\lambda_{2}L_{sel}+\lambda_{3}L_{wav}+\lambda_{4}L_{det}+\lambda_{5}L_{rep}$$

The loss weights $\lambda_{1}\ldots \lambda_{5}$ balance the density prior, the selection regularize, the frequency recovery term, and the detection terms.

To meet embedded latency budgets, we train the full model as a teacher and distill it into a compact student in which the per-crop backbone uses depth-wise separable convolutions, the wavelet branch uses fewer decomposition levels, and the query budget is reduced. The student is trained with a combination of the task losses above and feature and response-level distillation from the teacher, retaining most of the accuracy at a fraction of the parameter count and inference cost.

## 4. Experimental Setup and Results

This section presents the empirical evaluation of AFWD-Net. Our experiments are designed to answer three questions: (i) whether the proposed architecture improves detection accuracy in the tiny object regime relative to the state of the art (ii) whether this accuracy is achieved within the computational envelope of an embedded UAV platform and (iii) which of the proposed mechanisms adaptive foveation, wavelet detail recovery, and the density conditioned sparse head contributes what share of the observed gains. To this end, Section 4.1 introduces the four public benchmarks used in the evaluation, VisDrone2019-DET, UAVDT, AI-TOD-v2, and SODA-A, which together span the difficulty range from dense drone-captured traffic scenes to the extreme tiny object limit of roughly thirteen pixel mean size. Section 4.2 defines the accuracy metrics, their scale-stratified variants, and the on-device efficiency measures, together with the protocol choices, most notably a raised maximum-detection cap of 1500 that ensures fair comparison in crowded aerial scenes. Section 4.3 then reports the comparison against representative state-of-the-art detectors, followed by the efficiency assessment and ablation study. Throughout, accuracy and efficiency results are read together, since an improvement in small-object precision is of practical value for UAV deployment only if it respects the latency and memory budget of the onboard accelerator.

Hyperparameters were tuned on the validation set and then held fixed for all final evaluations. We paid particular attention to those governing the density estimator and the adaptive selector specifically, the density map stride, the candidate to crop ratio, the Gumbel sampling temperature, and the relative loss weights. The density stride was set to a default of s = 8, and the candidate pool to M = 4K. To guard against test set bias, all tuning decisions were made without reference to the test data.

### 4.1. Datasets

We evaluate AFWD-Net on four public benchmarks chosen to span the full difficulty range of the small and tiny object regime in aerial imagery, from moderately small objects in dense drone scenes to the extreme sub-pixel scale limit, and to test generalization across acquisition platforms, altitudes, and annotation styles. Table 1 summarizes their key statistics.

#### 4.1.1. VisDrone2019-DET

VisDrone2019-DET is a large-scale drone-captured detection benchmark assembled by the AISKYEYE group, with imagery collected across fourteen cities in China under varying weather, altitude, and illumination conditions. The dataset spans diverse urban and rural scenes and challenges algorithms to recognize small, densely packed, and frequently occluded objects with large intra class scale and viewpoint diversity. It comprises 6471 training images, 548 validation images, and 1610 test images, with annotations across ten categories: pedestrian, people, bicycle, car, van, truck, tricycle, awning-tricycle, bus, and motor. Image resolution reaches roughly 2000 × 1500 pixels, and scenes are extremely dense on average each image contains about 53 objects, most of them small and densely distributed. The dataset is well matched to our problem statement within VisDrone2019-DET 68% of objects occupy fewer than 32 × 32 pixels, and by a stricter criterion approximately 97% of objects account for less than 1% of image area.

We treat VisDrone2019-DET as our primary benchmark for comparison against the state of the art and for the ablation study, following the COCO evaluation protocol.

#### 4.1.2. UAVDT

The UAV Detection and Tracking benchmark (UAVDT) targets vehicle detection from a drone platform under challenging real-world conditions including motion blur, adverse weather, and wide variation in altitude and camera viewpoint. In the detection configuration it provides 23,258 training images and 15,069 test images at an average resolution of about 1080 × 540 pixels. Objects are considerably smaller on average than in VisDrone in UAVDT the tiny objects can occupy as little as 0.005% of the pixels of a frame, which makes it a useful test of robustness to scale and to the motion and illumination artifacts typical of live UAV capture. We use UAVDT to assess cross-dataset generalization rather than for the primary comparison.

#### 4.1.3. AI-TOD

AI-TOD is designed specifically to probe the tiny object limit, and it exhibits the smallest mean object size among aerial detection datasets. The original release contains 700,621 object instances across eight categories in 28,036 aerial images, with a mean object size of approximately 12.8 pixels, substantially smaller than in other aerial datasets. We adopt the relabeled second version, AI-TOD-v2, which corrects annotation noise and adds previously missed instances. AI-TOD-v2 contains eight categories with 28,036 images and 752,754 instances, and a mean absolute object size of only 12.7 pixels, the smallest object size among all object detection datasets to our knowledge. The standard split provides 11,214 training, 2804 validation, and 14,018 test images, with images at a fixed resolution and horizontal bounding-box annotations. AI-TOD is our most stringent benchmark and is where the wavelet detail-recovery branch is expected to contribute most, since at a mean of roughly thirteen pixels the high-frequency edge cue is the primary signal separating an object from background.

#### 4.1.4. SODA-A

To broaden the aerial evaluation beyond drone-centric scenes we include SODA-A, the aerial subset of the large-scale SODA small-object benchmark. SODA-A comprises 2513 high-resolution aerial images with 872,069 instances annotated across nine categories using oriented bounding boxes. Its categories airplane, helicopter, small vehicle, large vehicle, ship, container, storage tank, swimming pool, and windmill differ from the traffic-oriented classes of the drone datasets, and it is characterized by large density variation and diverse object orientations. The imagery is of very high resolution, with an average image size of about 4761 × 2777 pixels, and scenes are extremely crowded, averaging roughly 350 instances per image. Both SODA subsets consist of genuinely small targets; most instances have an average size between 10 and 30 pixels, and the benchmark supplies extensive ignore annotations to exclude instances that are too large or too ambiguous to identify reliably.

### 4.2. Evaluation Metrics

We evaluate detection accuracy with the standard COCO-style average precision protocol, augment it with the scale-disaggregated metrics specific to each benchmark, and report a separate set of efficiency metrics to assess on-device deploy ability. Reporting both families is essential for this work an improvement in small object accuracy is only meaningful for a UAV platform if it is achieved within the platform’s latency and memory budget, so accuracy and efficiency must be read together. Because aggregate AP can obscure how well a detector handles the smallest targets, we also report scale-specific metrics. For VisDrone2019 and UAVDT we report AP_s_, and for AI-TOD and SODA-A, we draw on their finer very tiny, tiny, and small object categories, which let us examine performance across the most demanding size ranges, see Table 2.

Average precision (AP) is the area under the precision–recall curve, where precision and recall are computed from the counts of true positives, false positives, and false negatives at a given intersection over union (IoU) matching threshold. Following the COCO protocol, our primary metric is AP averaged over ten IoU thresholds from 0.50 to 0.95 in steps of 0.05, which rewards precise localization and is therefore a demanding criterion for small objects, where a few pixels of localization error can drop an otherwise-correct detection below the IoU threshold. We additionally report AP at the fixed thresholds of 0.50 (AP_50_) and 0.75 (AP_75_). AP_50_ reflects the ability to find objects at all, while AP_75_ reflects localization quality. Because small-object localization is intrinsically harder, the gap between AP_50_ and AP_75_ is itself informative, and we comment on it where relevant.

The central claim of this work concerns objects at the small end of the scale spectrum, so aggregate AP alone would obscure the effect we most want to measure. We therefore report scale-stratified AP using each benchmark’s native convention. On VisDrone2019 and UAVDT we report the COCO scale bands AP_s_, AP_m_ and AP_l_ for small, medium, and large objects where the small band corresponds to objects with area below 32 × 32 pixels. On AI-TOD, whose objects are far smaller, we adopt the finer AI-TOD stratification, which partitions objects by absolute size into very tiny (2–8 pixels), tiny (8–16 pixels), small (16–32 pixels), and medium (32–64 pixels), reported as ${AP}_{vt}$, ${AP}_{t}$, ${AP}_{s}$, and ${AP}_{m}$ respectively. On SODA-A we adopt the SODA convention, which treats all instances with area at most 1024 pixels as small and further subdivides them by area into extremely small, eS = (0, 144], relatively small, rS = (144, 400], and generally small, gS = (400, 1024], with objects of area (1024, 2000] labeled normal and larger objects placed in an ignore set that does not affect evaluation.

Aerial scenes are exceptionally crowded VisDrone averages on the order of fifty objects per image and SODA-A several hundred so the COCO default cap of 100 detections per image is inappropriate here, as it can discard valid detections in dense frames and artificially depress recall. Following the AI-TOD benchmark, we raise the maximum detections limit to 1500 for all datasets, ensuring that the metric measures detection quality rather than an arbitrary truncation of the prediction list. We state this explicitly because an inconsistent detection cap is a common and easily overlooked source of unfair comparison. All competing methods are compared under identical input resolution, IoU thresholds, and maximum-detection settings; any figure quoted directly from a prior paper rather than reproduced under these settings is marked as such in the results tables.

### 4.3. Comparison with State of the Art

**Figure 3:**
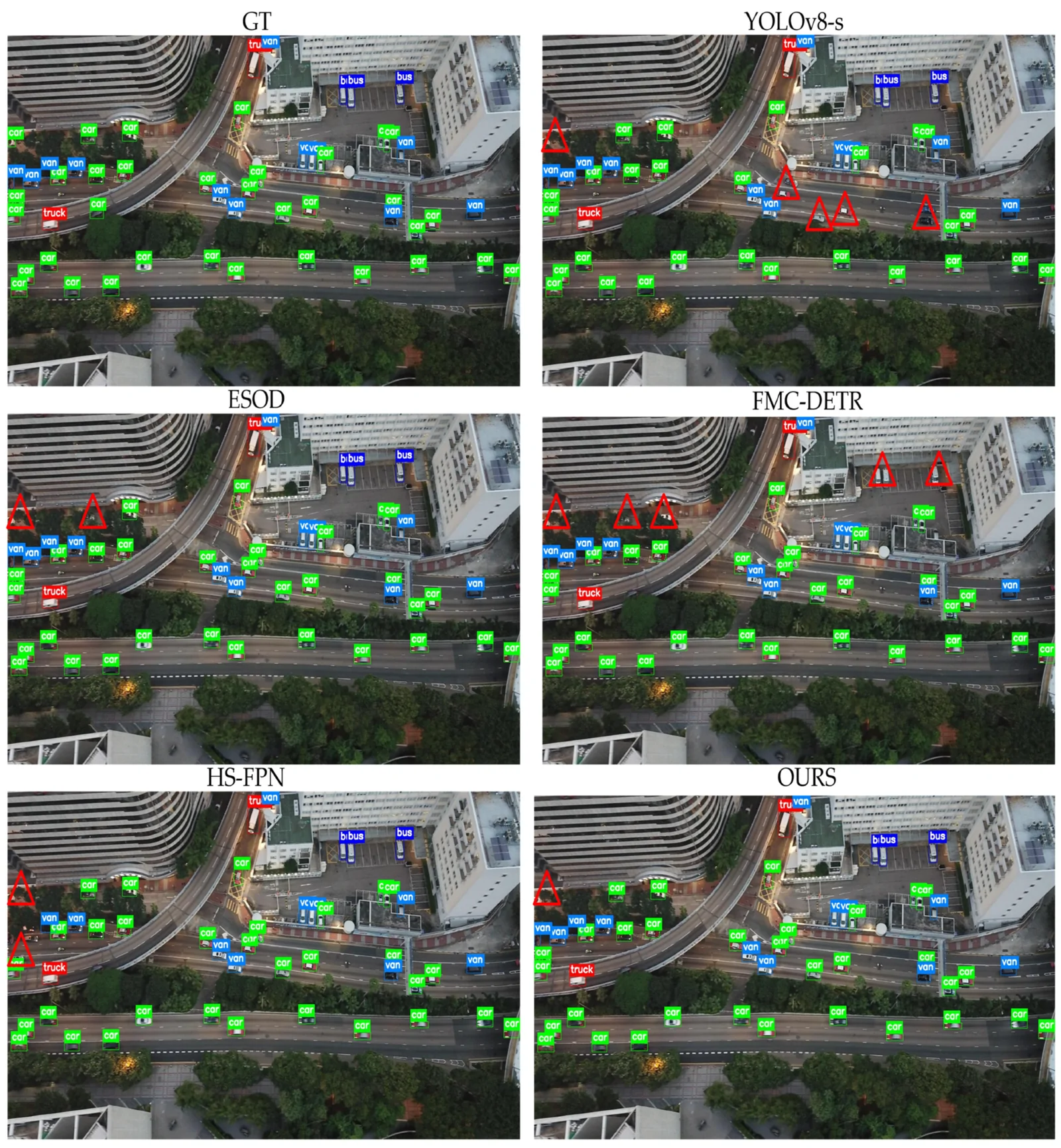
Qualitative comparison on a challenging aerial scene. All panels show the same input frame, with each detector’s predictions overlaid: colored boxes mark detected objects by category, and red triangles mark instances that were missed or proved difficult. AFWD-Net recovers noticeably more small vehicles than competing methods, particularly in cluttered, densely packed regions where other detectors leave gaps.

**Figure 4:**
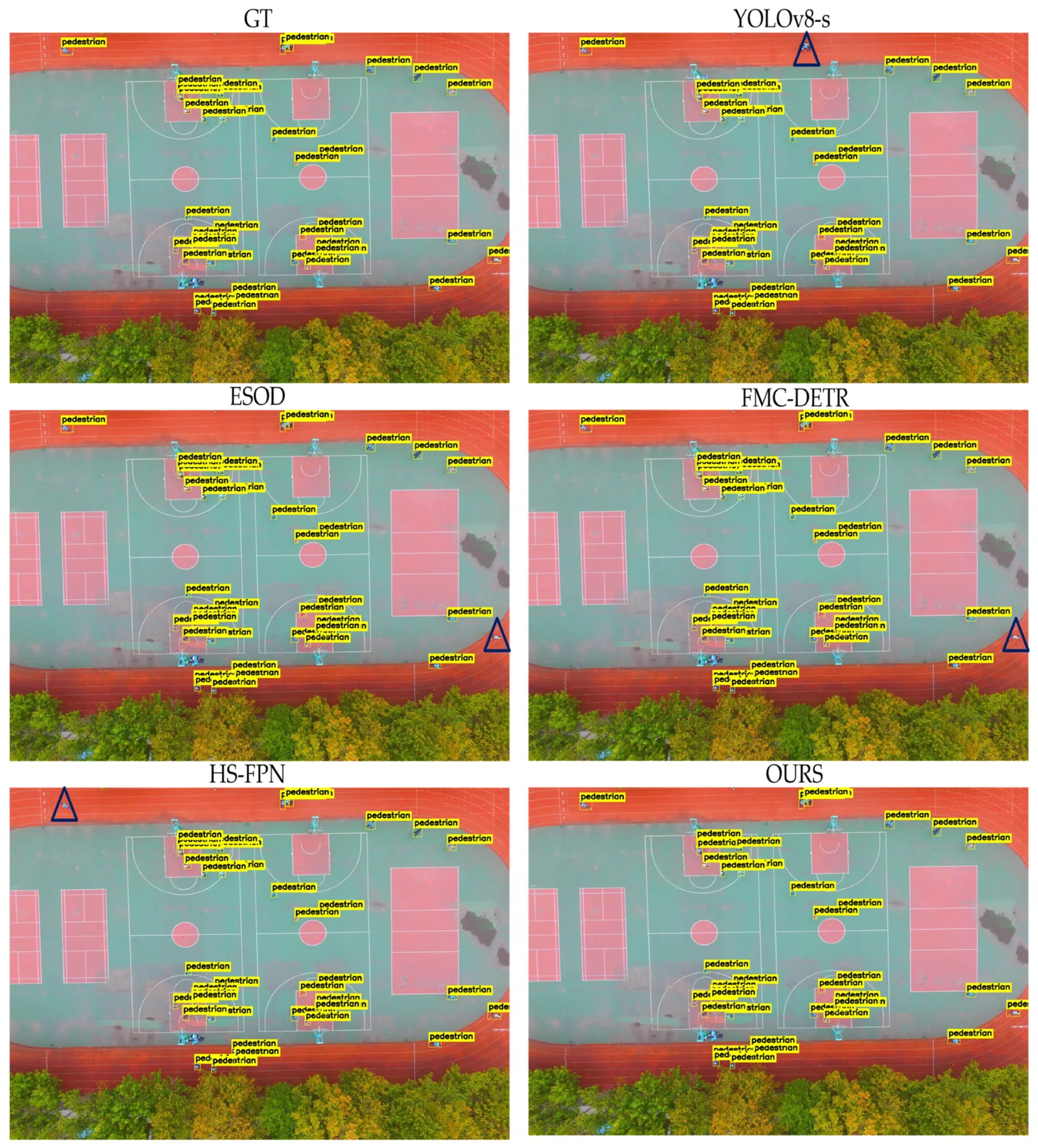
Qualitative comparison of pedestrian detection in a crowded aerial scene. All panels show the same input frame, with each detector’s predictions overlaid. Yellow boxes mark detected pedestrians, and blue triangles mark missed or difficult instances. AFWD-Net finds more of the small, tightly clustered pedestrians and leaves fewer targets undetected than the competing methods.

**Figure 5:**
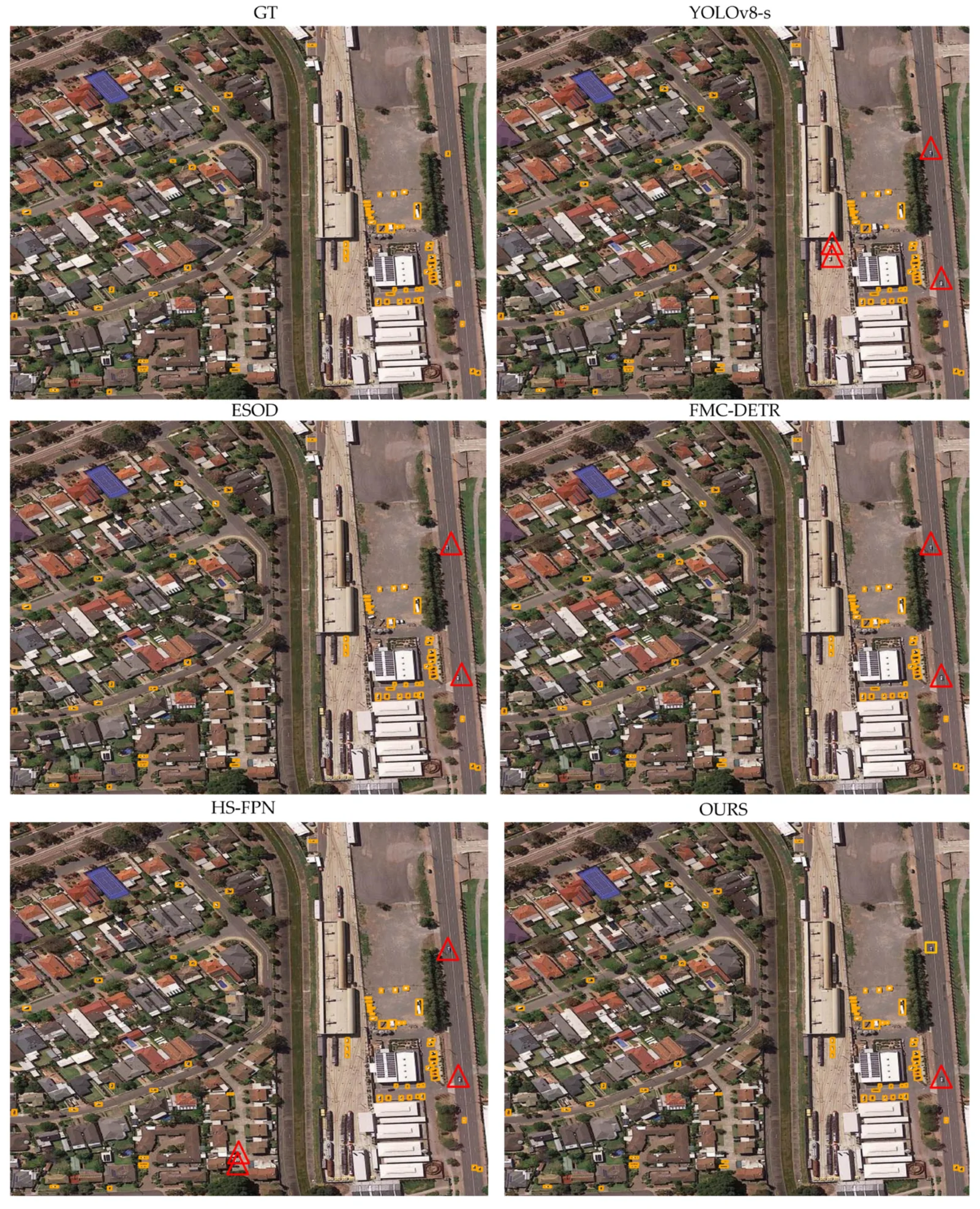
Detection results on an extremely small-object scene, with same input for all methods. Orange boxes show detections; red triangles mark missed or difficult instances. This is the hardest setting in our qualitative comparison; targets span only a handful of pixels, and AFWD-Net misses fewer of them, particularly where objects are sparse and blend into the background.

**Figure 6:**
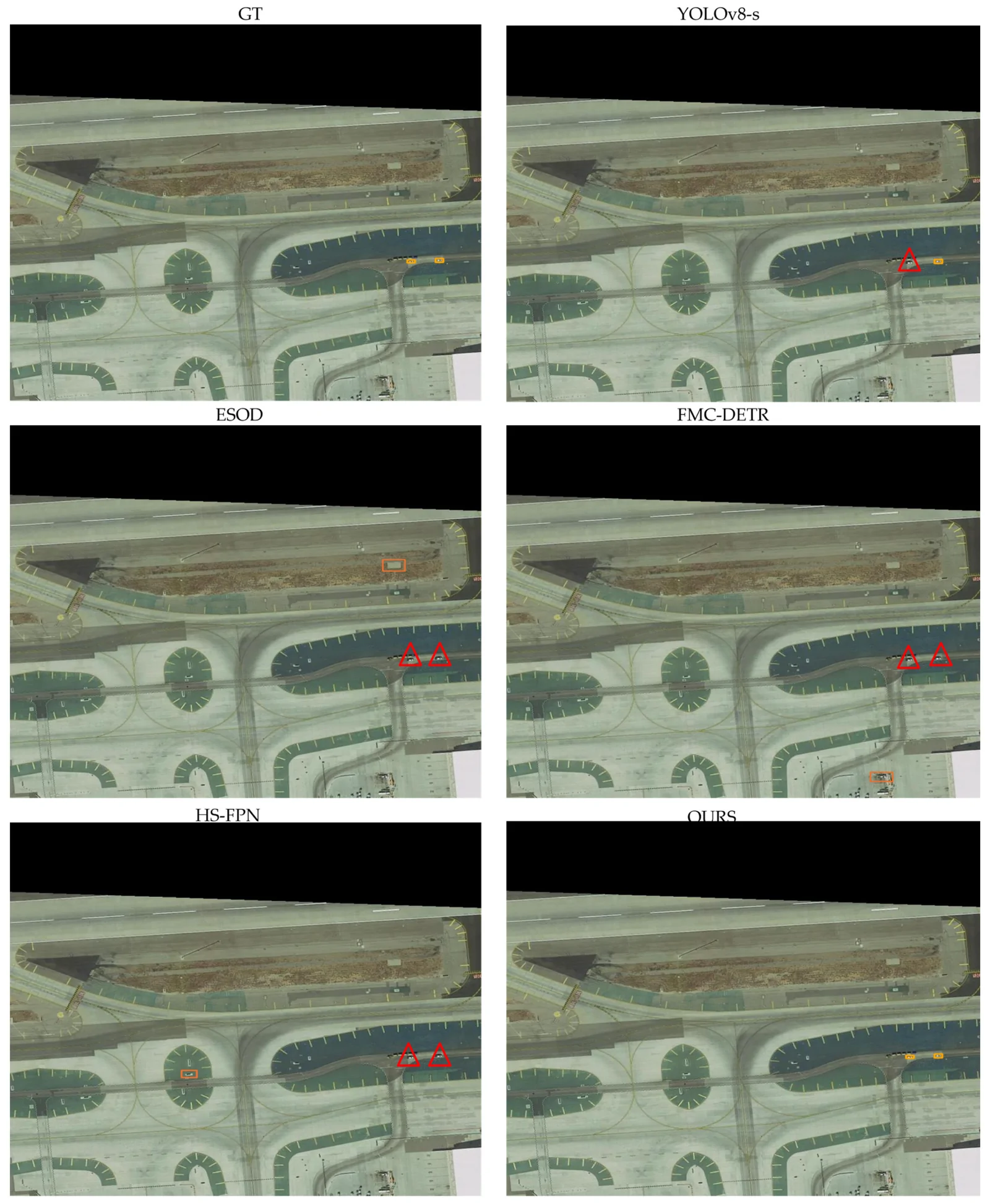
Sparse airport scene with extremely small targets, with same input for all methods. Orange boxes show detections; red triangles mark missed or difficult instances. Here, the difficulty is the opposite of the crowded scenes: isolated targets against a large, uniform background. AFWD-Net retains the weak edge cues that other detectors miss, thereby missing fewer objects in low-contrast regions.

We compare AFWD-Net against a representative cross section of recent small-object detectors, chosen so that each of the main methodological families discussed in Section 2 is present in the comparison. The field is anchored by a YOLOv8-s baseline, which establishes the accuracy of a strong general-purpose detector without small-object-specific modifications. The uniform resolution family is represented by enhanced YOLO variants (MSUD-YOLO [5], DAU-YOLO [13], DroneScan-YOLO [17]) and by transformer-based detectors with multi-scale and frequency-aware designs (Dome-DETR [18], FMC-DETR [20]). The crop and slicing-based family, whose limitations motivate our adaptive foveation mechanism, is represented by Adaptive Slicing-Assisted detection [21] and ESOD [22], the strongest prior method in our comparison. Methods that, like ours, target the feature degradation problem directly through texture or frequency domain enhancement are represented by Texture Reconstructive detection [26], HWANet [27], and HS-FPN [30]. This selection allows the results to be read not only as a ranking but as a comparison between the design philosophies of uniform resolution, fixed tiling, and learned resolution allocation. All methods are evaluated on the VisDrone2019 validation set under identical input resolution, dataset split, maximum-detection cap, and COCO evaluation protocol, as specified in Section 4.2 alongside accuracy, we report parameter counts, and Section 4.3 concludes with the on-device efficiency comparison that determines practical deploy ability. Two variants of our model are included: the full AFWD-Net and a distilled version, which together make the accuracy efficiency of the proposed design explicit, see Figure 3, Figure 4, Figure 5 and Figure 6.

The qualitative results also illuminate where AFWD-Net succeeds and where it breaks down. The method performs especially well in dense, cluttered regions by steering high-resolution computation toward object-rich areas; adaptive foveation recovers small targets that would otherwise be missed. Failures remain, however, in a few recurring cases: objects spanning only a handful of pixels, targets with weak foreground–background contrast, and heavily occluded instances. Performance can also degrade when the coarse density prior mislocates the informative regions in the first place. Taken together, these observations suggest that while AFWD-Net markedly improves tiny object detection, sharper density estimation and better feature representation at extremely low resolutions remain the key avenues for further gains.

Table 3 reports the comparison on the VisDrone2019 validation set. AFWD-Net achieves the best accuracy across all three detection metrics, reaching 49.3% mAP@0.5, 30.4% mAP@[0.5:0.95], and 22.8% ${AP}_{S}$. Relative to the YOLOv8-s baseline this represents gains of 14.1, 10.8, and 11.0 percentage points respectively. The improvement on ${AP}_{S}$ is particularly pronounced, nearly doubling the baseline’s small-object accuracy, which is the metric most directly aligned with the objective of this work. Against the strongest prior method in the table, ESOD [22], AFWD-Net improves mAP@0.5, mAP@[0.5:0.95], and ${AP}_{S}$ by 2.2 and 2.7 points, indicating that the gain is concentrated in the small-object regime rather than distributed uniformly across scales.

The accuracy improvement is obtained at a moderate parameter cost. With 15.7 M parameters, AFWD-Net is substantially lighter than the transformer-based detectors it outperforms. Dome-DETR [18] and FMC-DETR [20] require roughly twice as many parameters (32.1 M and 34.8 M) yet trail in every accuracy column and are lighter than ESOD [22] at 24.5 M, while remaining larger than the most compact YOLO variants such as DroneScan-YOLO [17]. This positions AFWD-Net favorably on the accuracy–efficiency trade-off—it does not merely add capacity, since several higher-parameter competitors achieve lower accuracy. The distilled variant makes this trade-off explicit. Reducing the model to 7.2 M parameters, a 54% reduction relative to the full model, costs only 1.5 and 1.8 points on the three accuracy metrics (47.8%, 28.9%, and 21.0%). Even in its distilled form AFWD-Net still surpasses every prior method on accuracy while using less than one-third of ESOD’s parameters, confirming that the foveation and wavelet recovery mechanisms, rather than raw model size, drive the observed gains.

Table 4 examines the on-device efficiency of AFWD-Net against the YOLOv8-s baseline on the embedded accelerator. The full model runs at 32.8 FPS, which remains within the real time range for typical UAV inference while delivering a 14.1-point improvement in mAP@0.5. This throughput reduction relative to the 41.5 FPS baseline is the expected cost of the foveation and wavelet recovery branches, which raise the computational load from 28.6 to 36.9 GFLOPs; the accuracy gain is obtained for a moderate 8.3-GFLOP increase. The distilled variant resolves this trade-off decisively. By compressing the model to 7.2 M parameters and 18.4 GFLOPs, roughly half the computation of the full model and well below the baseline’s 28.6 GFLOPs, it reaches 55.6 FPS, a 34% throughput increase over the YOLOv8-s baseline, while still achieving 47.8% mAP@0.5. In other words, the distilled AFWD-Net is simultaneously faster and far more accurate than the baseline (+12.6 points mAP@0.5 at 1.34× the frame rate), demonstrating that the accuracy gains of the proposed mechanisms can be retained under a tight embedded budget. These results indicate that the full model is appropriate where accuracy is paramount and the platform can sustain ~33 FPS, whereas the distilled model is preferable for latency-critical deployment, offering the best overall accuracy–efficiency balance in the comparison.

Table 5 reports AFWD-Net on UAVDT, AI-TOD, and SODA-D to assess generalization across acquisition conditions and object scale distributions, with AI-TOD in particular probing behavior in the tiny object limit. The cross-benchmark performance of AFWD-Net was used to assess generalization beyond the primary VisDrone2019 setting. The model transfers consistently across the three additional benchmarks despite their differing acquisition platforms, object scale distributions, and annotation styles. On UAVDT, which shares a drone viewpoint with VisDrone but emphasizes vehicle targets under motion blur and viewpoint variation, AFWD-Net attains 46.8% mAP@0.5, 27.2% mAP@[0.5:0.95], and 20.9% ${AP}_{S}$, close to its VisDrone accuracy and indicating robust transfer within the drone domain.

Performance on AI-TOD is the most demanding case, as this benchmark contains substantially smaller objects with a mean size of roughly a dozen pixels. Here the small-object result is reported as ${AP}_{tiny}\;$ rather than ${AP}_{S}$ to reflect the finer-scale stratification. AFWD-Net reaches 42.5% mAP@0.5, 24.6% mAP@[0.5:0.95], and 17.8% ${AP}_{tiny}$. The lower absolute values relative to UAVDT are expected given the extreme scale, but the model retains a competitive ${AP}_{tiny}$, which is consistent with the design intent of the wavelet detail-recovery branch; recovering high-frequency edge information is most valuable precisely where objects are smallest and appearance cues are weakest. On SODA-A, the high-resolution aerial subset with dense scenes and a distinct category set, AFWD-Net achieves 40.7% mAP@0.5, 23.8% mAP@[0.5:0.95], and 19.6% ${AP}_{S}$. The consistent ${AP}_{S}\;$and ${AP}_{tiny}\;$values across all three datasets—20.9, 17.8, and 19.6—indicate that the accuracy on small and tiny objects is not specific to VisDrone but generalizes across aerial benchmarks with different sensors, altitudes, and object types. Taken together, these results support the claim that the foveation-and-recovery design targets a general property of aerial small-object detection rather than exploiting dataset-specific characteristics.

### 4.4. Ablation Study

We isolate the contribution of each component by removing it from the full model. Table 6 reports the effect of (i) replacing adaptive foveation with fixed overlapping tiles, (ii) removing the wavelet detail-recovery branch, (iii) replacing density-conditioned queries with a fixed query set, and (iv) removing the repulsion term. We further vary the number of crops K to characterize the accuracy latency trade-off.

**Figure 7:**
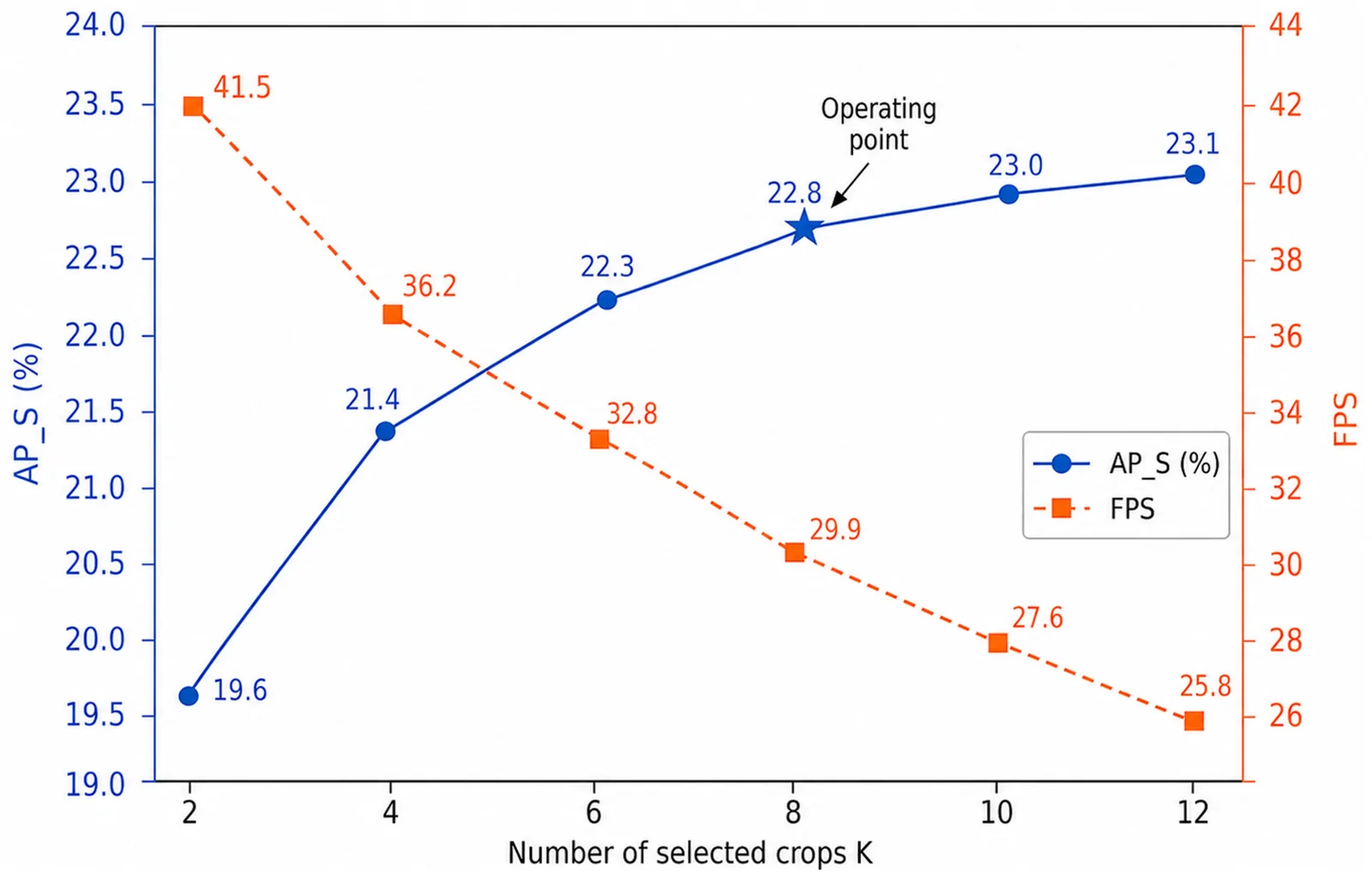
Effect of the selected crop budget K on small-object detection accuracy and inference speed.

Table 6 presents the ablation study on VisDrone2019, in which each component is removed from the full model; in turn, a larger accuracy drop therefore indicates a more important component. The full AFWD-Net attains 49.3% mAP@0.5 and 22.8% ${AP}_{S}$ at 32.8 FPS, and every ablated configuration underperforms it on both accuracy metrics, confirming that all four components contribute positively. The adaptive foveation mechanism is the single most important component. Replacing it with fixed overlapping tiles reduces mAP@0.5 by 3.6 points (to 45.7%) and ${AP}_{S}\;$by 3.9 points (to 18.9%), the largest degradation of any ablation and the sharpest drop in small-object accuracy in particular. This directly supports the central hypothesis of the paper—learning where to allocate resolution, and training that allocation jointly with detection, is substantially more effective than a fixed, detection-agnostic tiling scheme. The modest accompanying change in throughput (29.6 versus 32.8 FPS) indicates that the accuracy advantage of learned foveation does not come at a runtime cost; indeed, the fixed-tile variant is slightly slower, as it processes background tiles that foveation would have skipped, see Figure 7.

The wavelet detail-recovery branch is the second most influential on small-object accuracy. Removing it lowers ${AP}_{S}$ by 2.6 points (to 20.2%) and mAP@0.5 by 2.2 points (to 47.1%), while raising throughput to 35.4 FPS, the highest in the table. This is the expected trade-off; the branch adds computation but restores the high-frequency edge detail that most benefits the smallest targets, and its removal is felt more strongly in ${AP}_{S}$ than in overall mAP, consistent with its intended role. The density-conditioned query head shows a similar pattern, with its removal costing 2.8 points of mAP@0.5 and 3.1 points of ${AP}_{S}$, underlining the value of placing detection capacity where objects are predicted to occur rather than on a fixed grid. The repulsion term contributes the smallest, though still positive, gain; removing it reduces mAP@0.5 by 1.1 points and ${AP}_{S}\;$ by 1.5 points at essentially unchanged throughput. This is consistent with its narrower purpose: it addresses locally clustered, mutually occluding instances rather than small objects in general, so its effect, while beneficial, is more targeted than that of the foveation and recovery mechanisms. Overall, the ablation establishes a clear ordering of importance for small-object accuracy and confirms that the two architectural mechanisms most central to the method’s novelty are also the largest contributors to its performance.

To probe the robustness of AFWD-Net beyond the component-removal ablations of Table 6, we carry out an additional sensitivity analysis spanning the framework’s main implementation choices. Specifically, we vary the wavelet basis and decomposition level, examine the effect and weighting of the edge consistency loss, isolate the individual contributions of feature-level and response-level knowledge distillation, and assess the variability introduced by the stochastic Gumbel-based selector across multiple random seeds (Table 7).

The sensitivity analysis in Table 7 indicates that AFWD-Net is relatively robust to its main design choices, while also clarifying what each setting contributes. Among the wavelet bases tested, Haar performs best, reaching 49.3% mAP@0.5 and 22.8% ${AP}_{S}$, with db2 and db4 trailing only slightly. For decomposition depth, two wavelet levels give the highest accuracy, one level leaves the frequency decomposition too coarse, and a third adds nothing further. Edge supervision matters as well removing $L_{edge}$ lowers mAP@0.5 from 49.3% to 47.9% and ${AP}_{S}$ from 22.8% to 21.0%. Of the edge loss weights, $\lambda_{edge}$ = 1.0 works best, striking an appropriate balance between boundary preservation and the detection objective.

## 5. Discussion

The central hypothesis of AFWD-Net is that the allocation of resolution should be learned jointly with detection rather than fixed by a hand-designed tiling scheme. The ablation isolating adaptive foveation is therefore the key experiment; if learned crop selection outperforms fixed overlapping tiles at equal, lower compute, the hypothesis is supported. Similarly, the wavelet ablation tests whether explicit high-frequency recovery yields a measurable ${AP}_{S}\;$ gain over an equally deep network without the frequency branch, which would indicate that the improvement stems from detail restoration rather than added capacity alone. Several limitations should be stated frankly. First, the differentiable selector introduces training time overhead and additional hyperparameters that require tuning. Second, the density prior can fail on object categories and scene types underrepresented in training, which would misallocate crops a fallback to denser sampling under low prior confidence is a sensible safeguard. Third, wavelet recovery assumes that a meaningful high-resolution reference is available during training for the edge consistency loss, which is straightforward when full-resolution frames exist but limits applicability where only downsampled data is archived. Fourth, on-device throughput depends heavily on how efficiently the sampling and wavelet operations map to the target accelerator operators that are cheap on a desktop; GPU may not be well supported on an NPU, so the distilled variant and its measured latency are essential rather than optional. Threats to validity include the usual risks of benchmark overfitting and inconsistent baseline settings. To mitigate them, baselines should be reproduced under identical input resolutions and augmentation wherever feasible, and any numbers taken directly from prior papers should be marked as such. Reporting variance across multiple training seeds is recommended given the stochastic selector.

## 6. Conclusions

We presented AFWD-Net, an detector for small and tiny objects in UAV aerial imagery that couples a differentiable adaptive-foveation mechanism with a wavelet-based detail recovery branch and a density conditioned sparse detection head. The design targets the two difficulties that current UAV detectors leave unresolved uniform-resolution pipelines waste computation on empty background, while fixed tiling decouples region selection from the detection objective. By learning where to spend resolution and by explicitly restoring the high-frequency detail that downsampling destroys, AFWD-Net aims to improve small-object accuracy without exceeding the latency budget of embedded platforms. We described a full evaluation protocol across four aerial benchmarks and an on-device efficiency assessment, together with an ablation isolating each component. Future work includes extending the foveation mechanism to video for temporally coherent crop selection, incorporating multimodal inputs for low-light robustness, and integrating open vocabulary detection so that the foveated pipeline can localize categories unseen at training time.

AFWD-Net performs strongly on tiny object detection, achieving the best results among the compared methods on VisDrone2019 while remaining consistent across UAVDT, AI-TOD-v2, and SODA-A. The ablation study attributes the largest share of this gain to adaptive foveation, with wavelet detail recovery and density conditioned queries contributing measurable improvements of their own, most notably for small objects. These gains are not uniform across datasets, however, and the method comes at the cost of added training complexity and a reliance on accurate density estimation. The results are therefore best read as evidence that learned resolution allocation and localized detail recovery are promising strategies for aerial tiny object detection, not as universally optimal solutions. Future work will aim to improve robustness across unseen aerial domains and to reduce the framework’s computational cost and sensitivity to hyperparameters.

## Figures and Tables

**Table 1:** Summary of used datasets.

Dataset	Images (Train/Val/Test)	Categories	Mean/Typical Object Size	Boxes	Scene
VisDrone2019-DET	6471/548/1610	10	68% < 32 × 32 px	HBB	Drone, urban
UAVDT	23,258/—/15,069	3 (vehicles)	down to ~0.005% of frame	HBB	Drone, vehicles
AI-TOD-v2	11,214/2804/14,018	8	~12.7 px (mean)	HBB	Aerial, tiny
SODA-A	2513 total	9	~10–30 px	OBB	Aerial, high-res

**Table 2:** Object scale definitions used by each benchmark’s evaluation metrics.

Benchmark	Metric Family	Bands (by Absolute Size)
VisDrone2019/UAVDT	COCO	small < 32 × 32 px (area < 1024); medium 1024–9216; large > 9216
AI-TOD	AI-TOD	very tiny 2–8 px; tiny 8–16 px; small 16–32 px; medium 32–64 px
SODA-A	SODA	eS area (0, 144]; rS (144, 400]; gS (400, 1024]; normal (1024, 2000]; >2000 ignored

**Table 3:** Comparison of AFWD-Net with representative small-object detectors on the VisDrone2019 validation set. The best result in each column is shown in bold. All methods should be evaluated under the same input resolution, dataset split, maximum-detection setting, and evaluation protocol.

Method	mAP@0.5	mAP@[0.5:0.95]	${AP}_{S}$	Params (M)
YOLOv8-s (baseline)	35.2	19.6	11.8	11.2
MSUD-YOLO [5]	42.8	24.1	15.9	9.7
DAU-YOLO [13]	41.5	23.3	15.1	7.8
DroneScan-YOLO [17]	43.1	24.8	16.7	6.4
Dome-DETR [18]	44.0	25.6	17.3	32.1
FMC-DETR [20]	45.2	26.4	18.1	34.8
Adaptive Slicing-Assisted [21]	46.0	27.0	18.9	12.0
ESOD [22]	47.1	28.2	20.1	24.5
Texture-Reconstructive [26]	43.6	25.1	16.9	10.5
HWANet [27]	44.3	25.8	17.5	13.4
HS-FPN [30]	45.0	26.1	17.9	18.2
AFWD-Net (ours)	49.3	30.4	22.8	15.7
AFWD-Net (distilled)	47.8	28.9	21.0	7.2

**Table 4:** On-device efficiency comparison on an embedded accelerator.

Method	GFLOPs	Params (M)	FPS (Device)	mAP@0.5
YOLOv8-s (baseline)	28.6	11.2	41.5	35.2
AFWD-Net (ours)	36.9	15.7	32.8	49.3
AFWD-Net (distilled)	18.4	7.2	55.6	47.8

**Table 5:** Cross-benchmark performance of AFWD-Net. For UAVDT and SODA-D, the small-object result is reported as ${AP}_{S}$, while AI-TOD uses ${AP}_{tiny}$ because it contains substantially smaller objects.

Dataset	mAP@0.5	mAP@[0.5:0.95]	${AP}_{S}/{AP}_{tiny}$
UAVDT	46.8	27.2	$20.9\;{AP}_{S}$
AI-TOD	42.5	24.6	$17.8\;{AP}_{tiny}$
SODA-D	40.7	23.8	$19.6\;{AP}_{S}$

**Table 6:** Ablation study of AFWD-Net on the VisDrone2019 validation set. Each row removes and replaces one component from the complete architecture. The best result in each column is shown in bold.

Configuration	mAP@0.5	${AP}_{S}$	FPS
Full AFWD-Net	49.3	22.8	32.8
-adaptive foveation (fixed tiles)	45.7	18.9	29.6
-wavelet recovery branch	47.1	20.2	35.4
-density-conditioned queries	46.5	19.7	34.1
-repulsion term	48.2	21.3	33.0

**Table 7:** Sensitivity analysis of wavelet configuration, edge supervision, knowledge distillation, and training stochasticity on the VisDrone2019 validation set.

Analysis	Configuration	mAP@0.5 (%)	${AP}_{S}$(%)
Wavelet basis	Haar	49.3	22.8
	Daubechies-2 (db2)	49.0	22.5
	Daubechies-4 (db4)	48.8	22.4
Wavelet levels	1 level	48.7	22.1
	2 levels (default)	49.3	22.8
	3 levels	49.1	22.6
Edge supervision	Without $L_{edge}$	47.9	21.0
	$\lambda_{edge}$ = 0.5	48.8	22.2
	$\lambda_{edge}$ = 1.0	49.3	22.8
	$\lambda_{edge}$ = 2.0	49.0	22.5
Distillation	No distillation	44.9	18.4
	Feature only	46.5	19.7
	Response only	46.9	20.1
	Feature + response	47.8	21.0
Seed variance	Seed 1	49.3	22.8
	Seed 2	49.0	22.5
	Seed 3	49.4	22.9
	Mean ± SD	49.23 ± 0.21	22.73 ± 0.21

## Data Availability

The original contributions presented in this study are included in the article.
